# Sudden Sensorineural Hearing Loss after Orthopedic Surgery under Combined Spinal and Epidural Anesthesia

**DOI:** 10.1155/2016/4295601

**Published:** 2016-01-21

**Authors:** Ditza Vilhena, Luís Pereira, Delfim Duarte, Nuno Oliveira

**Affiliations:** ^1^Department of Otolaryngology & Head-Neck Surgery, Hospital Pedro Hispano, 4464-513 Matosinhos, Portugal; ^2^Department of Anesthesiology, Hospital São João, Porto, Portugal

## Abstract

Postoperative hearing loss following nonotologic surgery is rare. For patients undergoing subarachnoid anesthesia, the loss of cerebral spinal fluid and hence the drop in intracranial pressure can result in hearing loss and cranial nerve palsy. We report a case in which a patient sustained orthopedic surgery under combined spinal and epidural anesthesia complicated by severe and persistent sensorineural hearing loss. This report is a reminder that postoperative sudden sensorineural hearing loss is a poorly understood complication. A high index of suspicion is required for early diagnosis of this complication, although prompt treatment does not guarantee a good outcome.

## 1. Introduction

Postoperative sensorineural hearing loss (SNHL) following nonotologic surgery is rare and often detected only by audiometric evaluation [1–3]. This phenomenon has been reported following spinal anesthesia or lumbar puncture and can be unilateral or bilateral, typically affecting the low frequency range. A majority of patients' hearing deficits are transient, resolving without treatment within several days [2]. The loss of cerebral spinal fluid (CSF) and hence the drop in intracranial pressure transmitted to the inner ear perilymph via the cochlear aqueduct can result in SNHL [3]. We report a case in which a patient was submitted to orthopedic surgery under combined spinal and epidural anesthesia (CSEA) and complicated by severe and persistent SNHL.

## 2. Case Description

**Figure 1:**
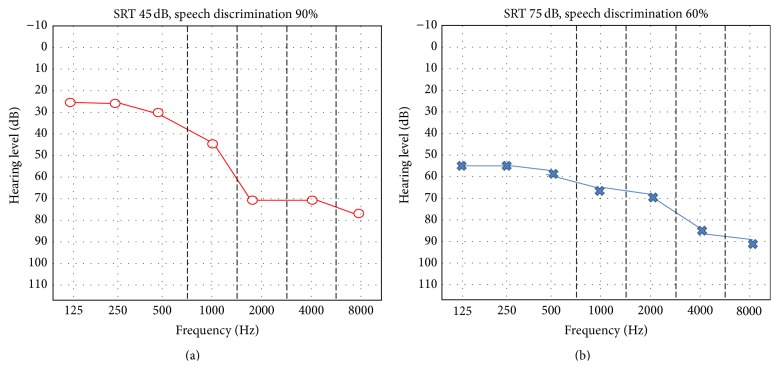
Initial audiogram, overlapping the audiogram 1 day before initiating hyperbaric oxygen therapy.

**Figure 2:**
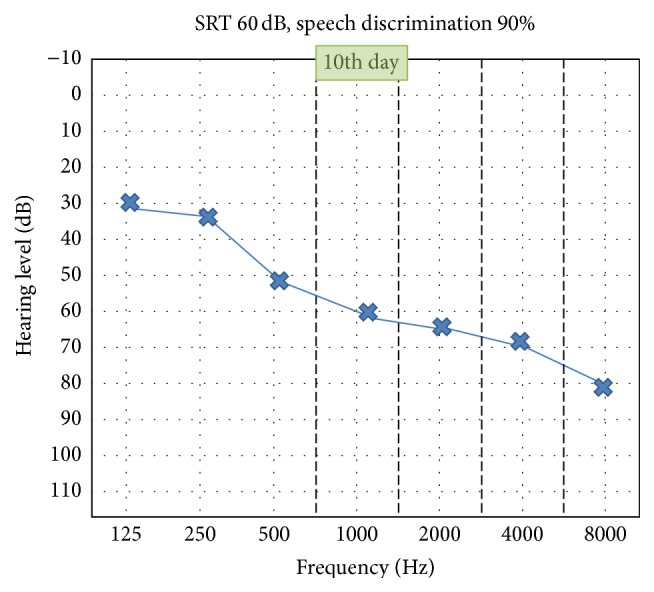
Audiogram after 10 sessions of hyperbaric oxygen therapy.

A 61-year-old man, with no relevant personal history, underwent a total knee replacement surgery under CSEA. After administration of lactated Ringer's solution (500 mL), an 18-gauge (G) epidural needle was inserted and loss of resistance technique was used to identify the epidural space followed by insertion of the 27 G pencil point spinal needle. When spontaneous backflow of CSF was observed, intrathecal 12.5 mg of heavy bupivacaine with 15 *μ*g fentanyl was injected into his subarachnoid space. CSEA has been uneventfully performed using needle through needle technique in the sitting position at L3-4 intervertebral space by midline approach at the first attempt. During surgery, an additional 500 mL of lactated Ringer's solution was administered. Surgery was completed within 100 minutes and was otherwise uneventful, and hemodynamic parameters remained within normal limits throughout the perioperative period. A unilateral left-sided SNHL was discovered shortly after completion of surgery in the recovery. This was associated with mild left-side tinnitus but no vertigo. There was no aural fullness, pain, headache, or postural element to his symptoms. Otoscopic examination was normal, Weber and Rinne tests suggested left-sided sensorineural hearing loss. A subsequent pure tone audiogram showed a bilateral SNHL, severe on the left side and moderate on the right side, and speech discrimination scores were 60% on the left and 80% on the right side (Figure 1). Additional laboratorial investigations were within normal limits. Magnetic resonance imaging scan was done and revealed no tumor or other abnormalities. The patient was kept restricted in bed in supine position. He was kept hydrated with plenty of oral and intravenous fluid. Systemic steroids were given to the patient and he had no substantial change in his hearing levels. Hyperbaric oxygen therapy treatment was started 10 days after the surgery and continued for 30 sessions. Significant improvement was noted, beginning after a few days; pure tone audiogram showed a left moderate SNHL, and speech discrimination score was 90% on the left side (Figure 2).

## 3. Discussion

Tinnitus combined with SNHL following either general or spinal anesthesia has been documented [1–3]. Hearing loss usually is minor and transient, but it can be permanent and disabling, particularly when associated with vertigo and tinnitus [3]. Vascular factors (thrombosis, spasm, emboli, and ischemia), autoimmunity, and viral disease of the cochlea are thought to be the main causes of idiopathic sudden SNHL [4]. However, mechanism of SNHL after spinal anesthesia is unclear, and it is suggested that a disruption of the endolymph/perilymph balance is caused by the decreased CSF pressure due to leakage of CSF through the dural puncture site. These decreases in perilymphatic pressure lead to the formation of endolymphatic hydrops, which displaces the hair cells on the basement membrane and results in low frequency hearing loss [5]. Loss of hearing for higher frequencies is more common in elderly male, possible because they may be more susceptible to the effects of subtle changes in inner ear pressure imbalance caused by CSF leakage [6]. This seems to be most likely the case in our patient that could have contributed to hearing loss. Cutting type needles of larger gauges (22 G) are associated with a higher incidence of this complication compared to finer gauge needles (25 G) or pencil point needles [7]. Although in our case we used a fine-bore needle, hearing loss was observed. We have found no report in the literature on SNHL after spinal anesthesia with a 27 G needle. Changes in blood volume and osmolarity during surgery might contribute to ischemia, and this may contribute to hearing loss [8]. In this case, blood pressure and pulse rate were normal during surgery, and the patient received sufficient liquid replacement. Treatment of SNHL after spinal anesthesia is controversial, and some patients may recover spontaneously [9]. Treatment options include maintaining supine position, hydration, cochlear vasodilators, systemic steroids, betahistine, hiperbaric oxygen therapy, epidural blood patches, plasma expanders, and carbogen inhalation, and results have been variable [3, 8]. Steroids, used in the present patient, reduce the inflammatory effect and endolymphatic pressure, and hyperbaric oxygen improves blood circulation [10–12] and reduces apoptotic phenomena. Muzzi et al. [13] showed that salvage hyperbaric oxygen therapy appeared to improve patients' pure tone hearing thresholds. An epidural blood patch may be used successfully to treat this complication, but it was not attempted in this case.

No substantial change in hearing levels was observed with the initial management (hydration, supine rest, and steroids). After initiation of hyperbaric oxygen therapy, hearing improvement was noted. At the end of the treatment, the patient's hearing loss improved from severe to moderate level and became almost equivalent to the contralateral side.

To the best of our knowledge, there are no prior accounts in the literature on the successful use of hyperbaric oxygen therapy in the treatment of SNHL following spinal anesthesia.

## 4. Conclusions

This report serves to remind clinicians that postoperative sudden SNHL is a poorly understood complication. A high index of suspicion is required for early diagnosis of this complication, although prompt treatment does not guarantee a good outcome. Since it is not possible at this time to anticipate which patients are at highest risk of developing SNHL after spinal anesthesia, prompt attention should be given to any patient complaining of hearing loss or tinnitus.

## References

[1] Schaaf H., Kampe S., Hesse G. (2004). Tinnitus after anaesthesia. *Anaesthesist*.

[2] Naesh O., Gilbert J., Makary A. (2005). Sensorineural hearing loss after general anaesthesia. *Anaesthesia and Intensive Care*.

[3] Kiliçkan L., Gürkan Y., Ozkarakas H. (2002). Permanent sensorineural hearing loss following spinal anesthesia. *Acta Anaesthesiologica Scandinavica*.

[4] Sara S. A., Teh B. M., Friedland P. (2014). Bilateral sudden sensorineural hearing loss: review. *Journal of Laryngology and Otology*.

[5] Michel O., Brusis T. (1992). Hearing loss as a sequel of lumbar puncture. *Annals of Otology, Rhinology and Laryngology*.

[6] Kiliçkan L., Gürkan Y., Aydin Ö., Etiler N. (2003). The effect of combined spinal-epidural (CSE) anaesthesia and size of spinal needle on postoperative hearing loss after elective caesarean section. *Clinical Otolaryngology and Allied Sciences*.

[7] Finegold H., Mandell G., Vallejo M., Ramanathan S. (2002). Does spinal anesthesia cause hearing loss in the obstetric population?. *Anesthesia and Analgesia*.

[8] Yildiz T. S., Solak M., Iseri M., Karaca B., Toker K. (2007). Hearing loss after spinal anesthesia: the effect of different infusion solutions. *Otolaryngology—Head and Neck Surgery*.

[9] Gültekin S., Özcan S. (2002). Does hearing loss after spinal anesthesia differ between young and elderly patients?. *Anesthesia and Analgesia*.

[10] Srinivasan B., Ethunandan M., Markus A. (2008). Sensorineural hearing loss after dental extraction under general anesthesia: report of a case. *Journal of Oral and Maxillofacial Surgery*.

[11] Stachler R. J., Chandrasekhar S. S., Archer S. M. (2012). Clinical practice guideline: sudden hearing loss. *Otolaryngology—Head and Neck Surgery*.

[12] Galanopoulos G., Rapti D., Nikolopoulos I., Lambidis C. (2011). Sudden sensorineural hearing loss after varicose vein surgery under general anesthesia. Case report. *Il Giornale di Chirurgia*.

[13] Muzzi E., Zennaro B., Visentin R., Soldano F., Sacilotto C. (2010). Hyperbaric oxygen therapy as salvage treatment for sudden sensorineural hearing loss: review of rationale and preliminary report. *Journal of Laryngology and Otology*.

